# Parsimonious continuous time random walk models and kurtosis for diffusion in magnetic resonance of biological tissue

**DOI:** 10.3389/fphy.2015.00011

**Published:** 2015-03-16

**Authors:** Carson Ingo, Yi Sui, Yufen Chen, Todd B. Parrish, Andrew G. Webb, Itamar Ronen

**Affiliations:** 1 Department of Radiology, C.J. Gorter Center for High Field MRI, Leiden University Medical Center, Leiden, Netherlands; 2 Department of Bioengineering, University of Illinois at Chicago, Chicago, IL, USA; 3 Department of Radiology, Northwestern University, Chicago, IL, USA

**Keywords:** anomalous diffusion, kurtosis, Mittag-Leffler function, continuous time random walk, fractional derivative, magnetic resonance imaging, stroke

## Abstract

In this paper, we provide a context for the modeling approaches that have been developed to describe non-Gaussian diffusion behavior, which is ubiquitous in diffusion weighted magnetic resonance imaging of water in biological tissue. Subsequently, we focus on the formalism of the continuous time random walk theory to extract properties of subdiffusion and superdiffusion through novel simplifications of the Mittag-Leffler function. For the case of time-fractional subdiffusion, we compute the kurtosis for the Mittag-Leffler function, which provides both a connection and physical context to the much-used approach of diffusional kurtosis imaging. We provide Monte Carlo simulations to illustrate the concepts of anomalous diffusion as stochastic processes of the random walk. Finally, we demonstrate the clinical utility of the Mittag-Leffler function as a model to describe tissue microstructure through estimations of subdiffusion and kurtosis with diffusion MRI measurements in the brain of a chronic ischemic stroke patient.

## 1. Introduction

In the first measurements of water diffusion in biological tissue using magnetic resonance imaging (MRI) systems, the term “apparent diffusion coefficient” (ADC) was chosen to highlight the fact that, although the free diffusion coefficient of water at body temperature is ~ 3 × 10^−3^ mm^2^/s, typical values in white matter (WM) and gray matter (GM) regions of interest in the human brain were found to be an order of magnitude smaller ~ 0.6 – 1.0 × 10^−3^ mm^2^/s due to hindrances imposed on water self-diffusion by the tissue microstructure [1–3]. Furthermore, it was recognized that the diffusion decay signal does not always follow a monoexponential decay as predicted by the Bloch-Torrey equation when diffusion weighted measurements are sampled at different time and length scales [4]. The non-monoexponential behavior suggested a superposition of two Gaussian diffusion populations with slow and fast ADC values which related to the volume fractions of intracellular and extracellular space within the imaging voxel, known as the biexponential model [5, 6]. Although the biexponential model provides a means to accurately fit the diffusion signal, the estimation of intracellular and extracellular volume fractions does not accurately correspond to the actual physical makeup of the underlying microstructure, either *in vivo* or even in a simple ghost erythrocytes model [7, 8].

As a result, more sophisticated geometrical models have been developed to more subtly identify diffusion properties that provide insight about tissue microstructure [9–13]. Some approaches describe the non-monoexponential signal as a shift from Gaussian diffusion to non-Gaussian diffusion. There have been efforts to utilize the exponential function and stretch the argument by a parameter in the exponent to provide an index that classifies the deviation from Gaussianity [14–17]. Additionally, there has been an attempt to characterize fixed biological tissue with higher moment analysis of the diffusion propagator in order to extract fractal-dimension measures [18, 19]. Another way to identify non-Gaussian diffusion, known as diffusional kurtosis imaging (DKI), uses a Taylor-series expansion of the argument in the exponential function in order to estimate excess kurtosis of the measured signal vs. the Gaussian case of a monoexponential decay [20]. However, due to the parabolic form of the fitting function in DKI, a limit must be placed on the maximum diffusion gradient strength for the fitting function to monotonically decrease with increased diffusion weighting [21]. It is possible to extend the gradient limit by including higher order terms in the expansion, however this requires the inclusion of terms greater than the second and fourth order cumulants that correspond to the second and fourth moments [22]. As very high strength gradients have recently become available, the mathematical form in DKI limits the ability to interrogate tissue microstructure [23]. Recently, the continuous time random walk (CTRW) has been applied to neural tissue to connect a mathematical model with a physical interpretation of diffusion [24–28]. As pointed out in Jensen and Helpern [21], the stretched exponential model in Bennett et al. [14] is not compatible with DKI as it is not an analytic function. However, by considering a model for subdiffusive processes, it is possible to compute the excess kurtosis using moment analysis of the Mittag-Leffler function (MLF). In CTRW theory, the MLF is a generalization of the exponential function, which is an analytic and monotonically decreasing function for all arguments. The MLF, while rigorously defined as a convergent power series, is, nevertheless, challenging to compute and fit for clinical MRI data, which is constrained in sampling due to practical limits on scan time. Here, we formulate simplified fitting forms of the MLF, connect subdiffusion to kurtosis, provide simulations of random walk conditions to illustrate the diffusion physics, and demonstrate measurements of non-Gaussian diffusion in the brain of a chronic stroke patient.

## 2. Materials and Methods

### 2.1. Continuous Time Random Walk Theory

The fundamental concept in CTRW theory is to extend the diffusion equation such that the fractional-order partial derivatives can be utilized as mathematical operators to describe the diffusion propagator, *P*(*x*, *t*):

(1) $$\frac{\partial^{\alpha }P(x,t)}{\partial t^{\alpha }}=D_{\alpha ,\beta }\frac{\partial^{\beta }P(x,t)}{\partial {|x|}^{\beta }},$$

where ∂*^α^*/∂*t^α^* represents the order of the Caputo fractional derivative in time for 0 < *α* ≤ 1, ∂*^β^*/∂|*x*|*^β^* represents the order of the Riesz fractional derivative in space for 1 < *β* ≤ 2, and *D_α_*_,_*_β_* is the generalized diffusion constant (length*^β^*/time*^α^*) [24, 29, 30].

The justification for making use of the fractional derivative operators is to provide a mathematical means to interpolate from homogeneous and relatively simple systems that exhibit local, Gaussian behavior to heterogeneous and relatively complex systems that exhibit non-local, power-law behavior [25, 30–34]. In the CTRW context, the fractional order operators, *α* and *β*, provide a description of a random walk’s likelihood to have broader distributions of waiting times and jump lengths, respectively, in comparison to classical Brownian motion. When *α* = 1 and *β* = 2, Equation (1) simplifies to the integer-order partial differential equation to describe Gaussian diffusion. Through the mean-squared displacement (MSD), when the ratio 2*α*/*β* < 1 the dynamics are sub diffusive and when 2*α*/*β* > 1 the dynamics are super diffusive [24, 25]. In the most general case in which *α* and *β* are of arbitrary orders, Equation (1) can be readily transformed to Fourier-Laplace space and represented in closed form as the MLF [32, 35]. The Fourier-Laplace transform, *P*(*x*, *t*)→ *p*(*q*, *s*), in Equation (1) is,

(2) $$p(q,s)=\frac{1}{s+D_{\alpha ,\beta }s^{1-\alpha }{|q|}^{\beta }}.$$

Applying the inverse Laplace transformation to Equation (2), we obtain a simple expression for the characteristic function (CF) of the diffusion propagator as,

(3) $$p(q,t)=E_{\alpha }[-D_{\alpha ,\beta }{|q|}^{\beta }t^{\alpha }],$$

where *E_α_* is the single-parameter MLF [36, 37]. When *α* = 1 and *β* = 2, Equation (3) becomes,

(4) $$p(q,t)=\exp(-D_{1,2}{|q|}^{2}t),$$

The MLF is an entire function defined as a power series expansion,

(5) $$f(z)=E_{\alpha }(z)=\sum_{k=0}^{\infty }\frac{z^{k}}{\Gamma (\alpha k+1)},$$

where the Γ function is the generalized form of the factorial function, defined for real numbers [38]. When *α* = 1, through the identity Γ (*k* + 1) = *k* !, Equation (5) becomes,

(6) $$f(z)=E_{1}(z)=\sum_{k=0}^{\infty }\frac{z^{k}}{k!},$$

which is the Taylor series definition of the exponential function.

For the case of time-fractional subdiffusion (i.e., 0 < *α* ≤ 1 and *β* = 2), in which the distribution of waiting times for the random walk follows power-law behavior (that is, more likely to wait longer times prior to each step), but the distribution of step lengths is Gaussian, Equation (2) becomes,

(7) $$p(q,s)=\frac{1}{s+D_{\alpha ,2}s^{1-\alpha }{|q|}^{2}},$$

and applying the inverse Laplace transform yields,

(8) $$p(q,t)=E_{\alpha }[-D_{\alpha ,2}{|q|}^{2}t^{\alpha }].$$

For the case of space-fractional superdiffusion (i.e., *α* = 1 and 1 < *β* ≤ 2), in which the distribution of waiting times is Gaussian, but the distribution for the random walker’s step lengths follows power-law behavior (that is, more likely to jump further), Equation (2) becomes,

(9) $$p(q,s)=\frac{1}{s+D_{1,\beta }{|q|}^{\beta }},$$

and applying the inverse Laplace transform yields,

(10) $$p(q,t)=\exp(-D_{1,\beta }{|q|}^{\beta }t).$$

### 2.2. Parsimonious Models for Diffusion Weighted MR Data

In order to write *D_α_*_,_*_β_* from Equation (3) in terms of the ADC with units of length^2^/time, *D*_1,2_,

(11) $$D_{\alpha ,\beta }\equiv D_{1,2}\frac{\tau^{1-\alpha }}{\mu^{2-\beta }},$$

where μ (e.g., μm) and *τ* (e.g., ms) are heuristic parameters that can be estimated to preserve the units for the diffusion coefficient, however, similar parameters have been for conservation of mass and heavy tailed limit convergence in fractal and fractional dynamics [33, 39–41]. When *α* = 1 and *β* = 2, Equation (11) shows that *D_α_*_,_*_β_* = *D*_1,2_. However, in order to provide a fitting estimate for Equation (3) from a diffusion MRI signal, at least five data points are required to find the *D*_1,2_, *α*, *β*, *μ*, and *τ*, which can be a challenging minimization problem and also time consuming for clinical *in vivo* diffusion MRI measurements. Since Equation (11) specifies *D*_1,2_, *μ*, and *τ* as a ratio, any number of parameter value combinations can satisfy successful fitting results. However, to constrain these parameters, initial conditions for *D*_1,2_, *μ*, and *τ* can be individually estimated using procedures described in Ingo et al. [24]. In order to simplify the fitting problem such that *D*_1,2_, *α*, *β* can be fitted without the need for additional parameters, *μ* and *τ*, we provide the following conversion to write Equation (3) in terms of the fitted diffusion weighting factor, *b*, defined as,

(12) $$b\equiv {\left(\gamma G\delta \right)}^{2}t,$$

where *γ* is the gyromagnetic ratio, *G* is the amplitude of the diffusion gradient, *δ* is the duration of the diffusion gradient pulse, and *t* is the effective diffusion time. The parameter *q* is defined as,

(13) q≡γGδ.

For the case of the spin-echo variant of the Stejskal-Tanner diffusion-weighted pulse sequence,

(14) $$t\equiv \Delta -\frac{\delta }{3},$$

where Δ is the time between diffusion gradient pulses, such that,

(15) $$b\equiv {\left(\gamma G\delta \right)}^{2}(\Delta -\frac{\delta }{3})=q^{2}t.$$

Combining Equations (3) and (11),

(16) $$p(q,t)=E_{\alpha }[-D_{1,2}\frac{\tau^{1-\alpha }}{\mu^{2-\beta }}q^{\beta }t^{\alpha }],$$

which can also be written as,

(17) $$p(q,t)=E_{\alpha }[-D_{1,2}\frac{\tau^{1-\alpha }}{\mu^{2-\beta }}{\left(q^{2}\right)}^{\beta /2}t^{\alpha }].$$

By substituting *q*^2^ with *b*/*t*, Equation (17) becomes,

(18) $$p(\frac{b}{t},t)=E_{\alpha }[-D_{1,2}\frac{\tau^{1-\alpha }}{\mu^{2(1-\beta /2)}}{\left(\frac{b}{t}\right)}^{\beta /2}t^{\alpha }],$$

which can be rearranged as,

(19) $$p(\frac{b}{t},t)=E_{\alpha }[-D_{1,2}\frac{\tau^{1-\alpha }}{\mu^{2(1-\beta /2)}}{\left(b\right)}^{\beta /2}t^{\left(\alpha -\beta /2\right)}],$$

and the substitution can be made to define the classical diffusion coefficient, *D*, raised to the order *β*/2 as,

(20) $$D^{\beta /2}\equiv D_{1,2}\frac{\tau^{1-\alpha }}{\mu^{2(1-\beta /2)}}t^{\left(\alpha -\beta /2\right)},$$

in which *D^β^*^/2^ has units, for example, (mm^2^/s)*^β^*^/2^, resulting in the form,

(21) $$p(b)=E_{\alpha }[-{\left(bD\right)}^{\beta /2}],$$

such that *D*, *α*, and *β* can be estimated from a minimum of three data samples. Equation (21) is a compact equation with which to interrogate diffusion and perfusion properties in biological tissues in the realms of sub-, super-, and Gaussian diffusion to capture, for example, intravascular incoherent motion (IVIM) at small *b*-values [42]. However, if one is interested in capturing only subdiffusion and Gaussian diffusion properties of WM and GM of the brain, which was demonstrated to be the general behavior in Ingo et al. [24] as *β* → 2 and 2*α*/*β* ≤ 1 for most voxels, Equation (21) can be further condensed by setting *β* = 2,

(22) $$p(b)=E_{\alpha }(-bD).$$

As an alternative approach to develop a model for subdiffusion, Equations (8) and (11) can be combined by setting *β* = 2 to give,

(23) $$p(q,t)=E_{\alpha }[-D_{1,2}\tau^{1-\alpha }{|q|}^{2}t^{\alpha }],$$

which for estimated values of *τ* ≃ *t*, as demonstrated in Ingo et al. [24], produces an effective mathematical form given by the two parameter model in Equation (22) such that the equations are interchangeable under the condition *τ* ≃ *t*.

### 2.3. Connecting Subdiffusion to Excess Kurtosis

Qualitatively, kurtosis is a measure to non-specifically describe the peakedness and/or heavy tail shape in a probability distribution via the standardized fourth moment [43]. For example, a Gaussian probability density function (pdf) has a kurtosis value of 3, whereas the hyperbolic secant pdf has a kurtosis value of 5 as its shape is both more peaked and heavier-tailed than the Gaussian shape. A convenient index called excess kurtosis is defined as the difference between the estimated kurtosis value for a given distribution with the value of the Gaussian pdf (i.e., 3), such that the excess kurtosis of a Gaussian pdf is 0. In applications of diffusion MRI, approaches have been developed to expand the logarithm of the exponential signal decay in the form of a Taylor series to arrive at higher order diffusion models [20, 44, 45]. Specifically, in Jensen et al. [20], the concept of excess kurtosis was connected to the Taylor series expansion of *b*, which, in its simplest scalar form, is given by,

(24) $$S/S0=\exp(-bD+\frac{1}{6}b^{2}D^{2}K_{\mathrm{app}}),$$

where *K_app_* is the apparent excess kurtosis. When *K_app_* = 0, the second term in Equation (24) disappears resulting in a monoexponential form, which is the CF for a Gaussian pdf. In Jensen et al. [20], *in vivo* diffusion measurements of the human brain have shown *K_app_* ≥ 0, indicating non-Gaussian dynamics. In the context of the mathematical definition of the true excess kurtosis, *K_t_*, these measurements indicate that the diffusion of water in neural tissue follows probability distributions with standardized fourth moments which are broader than the Gaussian case,

(25) $$K_{t}\equiv \frac{\langle x^{4}\rangle }{{\langle x^{2}\rangle }^{2}}-3.$$

Due to the parabolic form of the argument in Equation (24), there is a limit on the maximum *b*-value which can be fitted using this model. For typical diffusion MRI measurements in the human brain, the maximum values range from *b* ≤ 2000 – 4000 s/mm^2^ in order for the fitting function to monotonically decrease with increased diffusion weighting, as detailed in Jensen and Helpern [21]. In contrast to the parabolic form of Equations (24), (3), (8), (10), and (21) monotonically decrease as *b*→∞. However, Equations (3), (10), and (21) cannot be analytically expanded at *q* = 0 when *β* < 2 and, so, the second moment diverges for the diffusion propagator, *P*(*x*, *t*), which by extension, also applies to the stretched exponential models (about *q* or *b*) in Bennett et al. [14], Hall and Barrick [15], Magin et al. [16], Palombo et al. [17]. Although the second moment for these models diverge, it has been shown that rescaling methods lead to a pseudo MSD that is proportional to a trajectory that is faster than the Gaussian case of linear time dependence [25, 26]. On the other hand, Equations (8) and (23) can be expanded as analytic functions of *q* in order to compute the higher order moments and estimate the excess kurtosis as shown below.

By first utilizing the simple form of the Laplace-Fourier solution to time-fractional subdiffusion in Equation (7), the MSD can be computed by taking the second derivative of Equation (7) with respect to *q* in the limit of *q* → 0 and then performing a Laplace inversion,

(26) $$\langle x^{2}(t)\rangle =L^{-1}\lim_{q\to 0}\{\frac{d^{2}p(q,s)}{dq^{2}}\},$$

which gives,

(27) $$\langle x^{2}(t)\rangle =L^{-1}\{2D_{\alpha ,2}s^{-(\alpha +1)}\},$$

and utilizing the common Laplace and time domain transform pair,

(28) $$\frac{t^{\alpha }}{\Gamma (\alpha +1)}=L^{-1}\{s^{-(\alpha +1)}\},$$

yields the form of the MSD,

(29) $$\langle x^{2}(t)\rangle =\frac{2D_{\alpha ,2}}{\Gamma (\alpha +1)}t^{\alpha },$$

as reported in Metzler and Klafter [25]. Extending the formalism of operating in the Laplace domain, the fourth moment of Equation (7) can be expressed as,

(30) $$\langle x^{4}(t)\rangle =L^{-1}\{24{\left(D_{\alpha ,2}\right)}^{2}s^{-(2\alpha +1)}\},$$

and inverting into the time domain using the transform in Equation (28) gives,

(31) $$\langle x^{4}(t)\rangle =\frac{24{\left(D_{\alpha ,2}\right)}^{2}}{\Gamma (2\alpha +1)}t^{2\alpha }.$$

Inserting Equations (29) and (31) into Equation (25), the excess kurtosis of the MLF for time-fractional subdiffusion is,

(32) $$K_{\mathrm{MLF}}=6\frac{\Gamma^{2}(\alpha +1)}{\Gamma (2\alpha +1)}-3.$$

**FIGURE 1:**
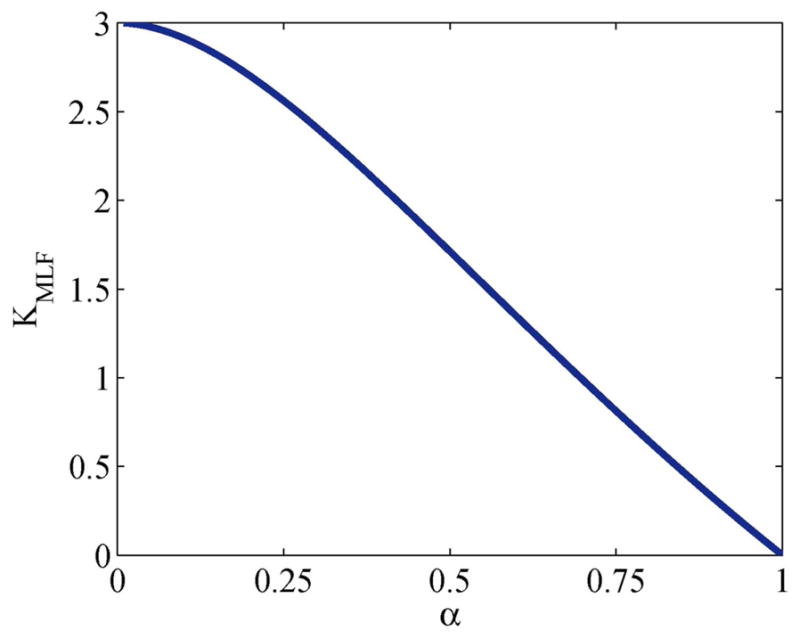
Plot of Equation (32) for the kurtosis, *K_MLF_*, computed in the Mittag-Leffler representation of subdiffusion vs. time-fractional derivative, *α*.

When *α* = 1, the MLF becomes the monoexponential CF and *K_MLF_* = 0. For 0 < *α* < 1, *K_MLF_* > 0 with the maximum excess kurtosis value limited to max(*K_MLF_*) = 3 when *α* → 0. Equation (32) is plotted in Figure 1 which shows a nearly inverse linear relationship between *K_MLF_* and *α*. Therefore, the monotonicity of the MLF provides an alternative and explicit means to interrogate the kurtosis of the pdf for the diffusion propagator, but with the important advantage that there are no longer limitations on the *b*-value that can be fitted, as is the case for Equation (24).

Interestingly, the form in Equation (32) coincides with the universal scaling law derived in Goychuk et al. [46], lim

(33) $$\lim_{t\to \infty }\frac{\langle x{\left(t\right)}^{2}\rangle }{{\langle x(t)\rangle }^{2}}=2\frac{\Gamma^{2}(\alpha +1)}{\Gamma (2\alpha +1)}-1,$$

which gives the relationship of the mean particle position to its MSD for subdiffusive diffusion dynamics described by *α*. These authors show through numerical simulations that the relationship in Equation (33) holds, within statistical error, under varying values of applied forces and temperatures, as is clearly illustrated in Figure 2 of Goychuk et al. [46]. Furthermore, a similar result to Equation (33) was derived in He et al. [47], showing this relationship is also a measure of ergodicity breaking behavior. Therefore, it is possible that Equations (32) and (33) reflect fundamental properties of biological tissue structure, which are minimally impacted by temperature and pressure, for diffusion MRI studies.

### 2.4. Random Walk Monte Carlo Simulations

**FIGURE 2:**
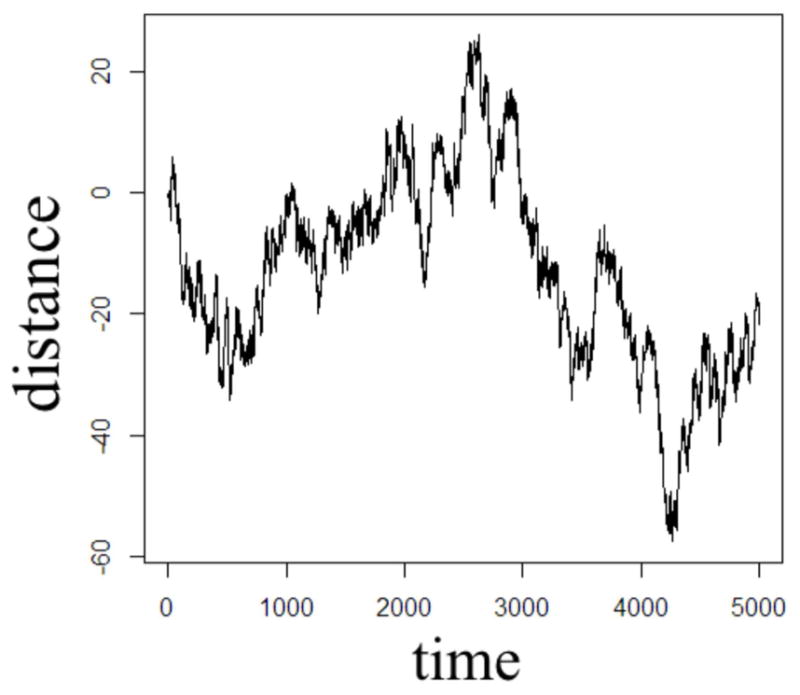
One dimensional random walk simulation of Brownian motion given by a Gaussian distribution of the waiting time parameter (*α* = 1) and a Gaussian distribution of the jump length parameter (*β* = 2).

**FIGURE 3:**
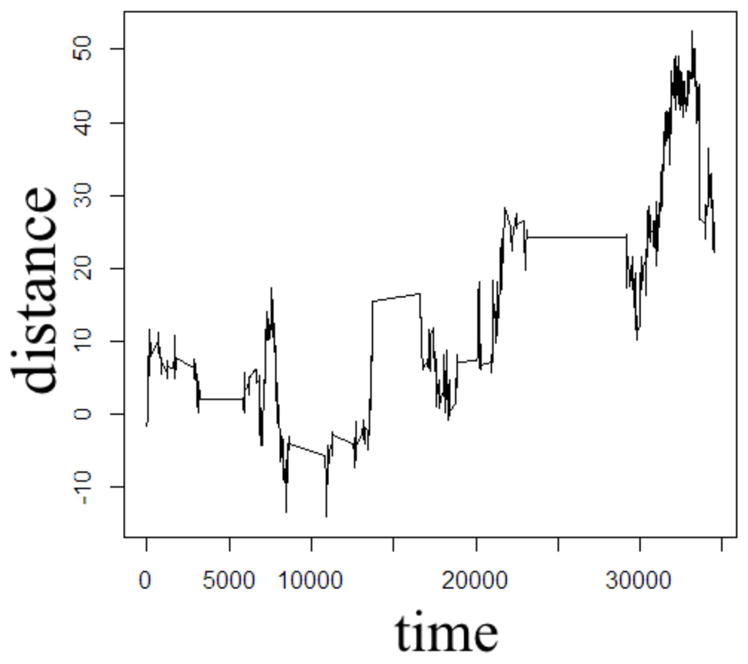
One dimensional random walk simulation of time-fractional subdiffusion given by a power-law distribution of the waiting time parameter (*α* = 0.75) and a Gaussian distribution of the jump length parameter (*β* = 2).

**FIGURE 4:**
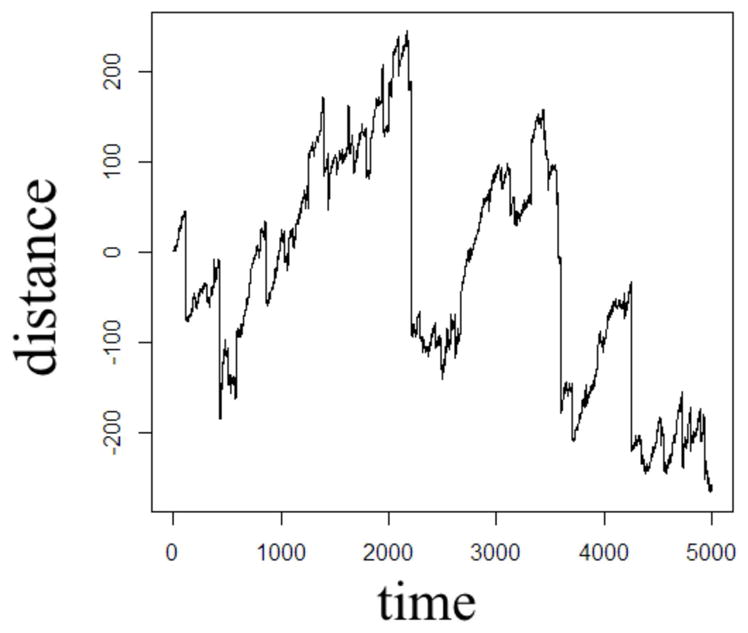
One dimensional random walk simulation of space-fractional superdiffusion given by a Gaussian distribution of the waiting time parameter (*α* = 1) and a power-law distribution of the jump length parameter (*β* = 1.5).

**FIGURE 5:**
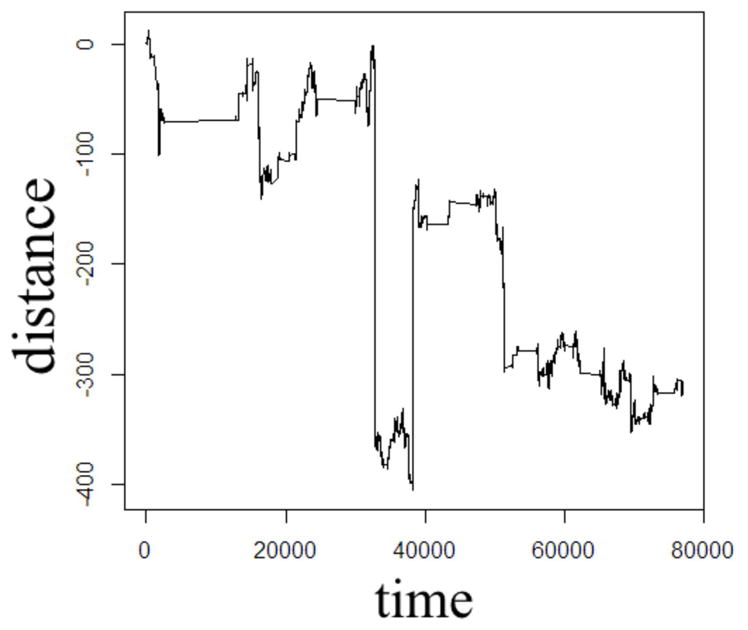
One dimensional random walk simulation of space- and time-fractional anomalous diffusion given by a power-law distribution of the waiting time parameter (*α* = 0.75) and a power-law distribution of the jump length parameter (*β* = 1.5).

In order to illustrate the diffusion dynamics specified in Equations (3), (4), (8), and (10), one-dimensional random walk simulations were performed using the R software environment [48]. These numerical simulations provide a context to demonstrate how the order of the fractional derivatives in Equation (1) impact the governing statistics in a random walk’s distributions of waiting times and jump lengths. To simulate the sample path as governed by the CF in Equation (4), a walk with independent and identically distributed (iid) Gaussian jump lengths and iid Gaussian waiting time increments produces the dynamics described by Brownian motion in Figure 2. To simulate the sample path as governed by the CF in Equation (8), a walk of iid Gaussian step lengths, but with iid power-law waiting time increments, produces the dynamics described by time-fractional subdiffusion in Figure 3 with *α =* 0.75. To simulate the sample path as governed by the CF in Equation (10), a walk of iid power-law jump lengths and iid Gaussian waiting time increments produces the dynamics described by space-fractional superdiffusion in Figure 4 with *β =* 1.5. To simulate the sample path as governed by the CF in Equation (3), in a walk with step lengths and waiting times that are both independently iid power-law produces the dynamics described by time- and space-fractional diffusion in Figure 5 with *α =* 0.75 *β =* 1.5. In order to compute the iid power law behavior for the waiting times were selected from the Pareto distribution,

(34) $$F(t)={\left(\frac{t}{c}\right)}^{-\frac{1}{\alpha }},$$

and the zero-mean corrected Pareto distribution for step lengths in the positive and negative directions,

(35) $$F(x)={\left(\frac{x}{c}\right)}^{-\frac{1}{\beta }}-\frac{\beta }{\beta -1}c^{\frac{1}{\beta }},$$

where *c* is a constant chosen to be 1 for these simulations to readily demonstrate the square root relationship between distance and time for diffusion, and *t* and *x* have arbitrary units with *t* ≥ 1 and |*x*| ≥ 1. The R codes were adapted from the examples fully described in chapter 5 of Meerschaert and Sikorskii [34].

### 2.5. Diffusion MRI Experiments

**FIGURE 6:**
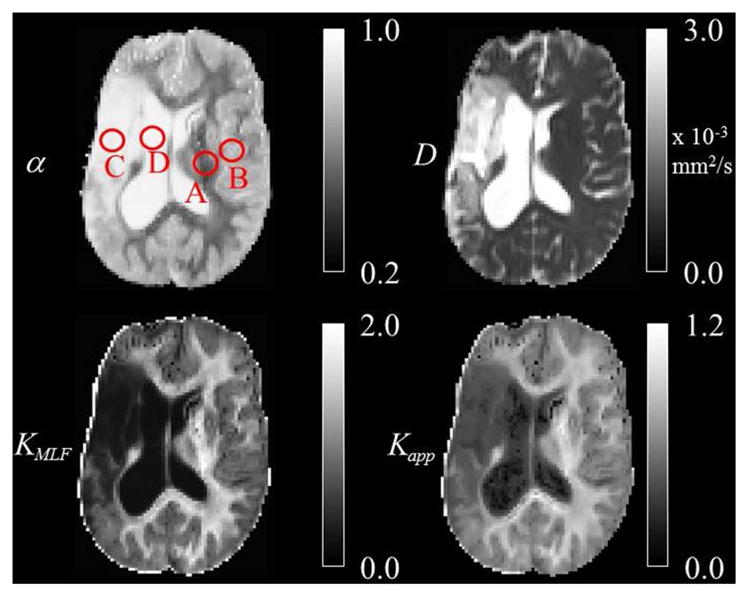
Trace parameter maps of *α*, *D*, *K_MLF_*, and *K_app_* for an axial slice through a brain of a chronic stroke patient with ROIs in the (A) WM, (B) GM, (C) IT, and (D) CSF.

One patient with chronic ischemic stroke was scanned on a 3 Tesla Siemens Trio MRI scanner with a 16 channel transmit/receive head coil (SiemensMedical Solutions, Erlangen, Germany). The imaging protocol was approved by Institutional Review Board at Northwestern University. Diffusion-weighted spin-echo echo planar imaging (SE-EPI) experiments were performed with the following pulse sequence parameters: echo time *TE* = 102 ms, repetition time *TR* = 6 s, Δ = 41.2 ms, *δ* = 30.6 ms, *b*-values = 0, 500, 1000, 3000, 4000 s/mm^2^, 3 orthogonal diffusion weighted directions, number of averages *NA* = 6, inplane voxel resolution = 2 × 2 mm, voxel thickness = 4mm, 20 axial slices, scan time ~ 6 min. The raw diffusion weighted data were corrected for Rician noise by estimating the variance (*σ*^2^) in the signal intensity of the ventricle at each b-value, such that $S_{rn}=\sqrt{S^{2}-2\sigma^{2}}$, which is a limited approach in assuming the spatial distribution of the noise is homogeneous in this multi-channel acquisition [49, 50]. The Rician noise-corrected diffusion weighted images were skull-stripped utilizing the Brain Extraction Tool of the FMRIB Software Library [51]. All skull-stripped and Rician noise-corrected diffusion weighted images were co-registered to the *b =* 0 image space using Statistical Parametric Mapping (SPM8, Wellcome Department of Cognitive Neurology, London, UK, http://www.fil.ion.ucl.ac.uk/spm). Using the Levenberg-Marquardt minimization algorithm in Matlab (Mathworks, Natick, MA), the averages of the 3 diffusion weighted direction data were fitted on a voxel-wise basis to Equation (24) and Equation (22) with the MLF algorithm in Podlubny [52], Gorenflo et al. [53]. Following estimations of *D* and *α*, the excess kurtosis, *K_MLF_* was computed using the conversion provided in Equation (32). Additionally, data from *b =* 0 and *b =* 1000 s/mm^2^ were fitted to Equation (4) in order to estimate the classical ADC for comparison to the estimations of *D* using Equations (24) and (22). The isotropic parameter maps of *D* as estimated by the MLF, *α*, *K_MLF_*, and *K_app_* for the same axial slice through the stroke patient’s brain are shown in Figure 6. Using the same procedures, Equations (24) and (22) were also fit to *b*-values = 0, 500, 1000, 3000 s/mm^2^ to consider the change in kurtosis estimates by removing the *b =* 4000 s/mm^2^ data.

## 3. Results

The Monte Carlo simulations in Figures 2–5 can be interpreted as the random motion of a single particle governed by the statistical conditions imposed by the order of the fractional derivatives in the generalized diffusion equation in Equation (1). Qualitatively, it can be observed that the magnitude of the distances in the sample paths are on the order of square-root of the time duration, a characteristic which is particularly evident in the Brownian motion case of Figure 2. For the case of time-fractional subdiffusion in Figure 3, the sample path clearly evolves slower than the square-root of time, whereas for space-fractional superdiffusion in Figure 4 the particle diverges faster than the Brownian case over time. Interestingly, for the time- and space-fractional diffusion in Figure 5 in which the simulation conditions have a ratio of 2*α*/*β =* 1, the sample path distance roughly evolves with the square root of time, however, the trajectory is clearly different from the Brownian motion simulated in Figure 2. Experimentally, the case for Brownian motion in Figure 2 is readily distinguishable from the time- and space-fractional diffusion scenario in Figure 5 by inspection of the CFs with respect to the monoexponential form.

In Figure 3, the apparent subdiffusive behavior of the particle appears as Brownian motion within certain time intervals, but is also punctuated by long periods in which the particle makes little or no random movement as a consequence of the heavy-tailed likelihood to wait longer than in the Gaussian case. In the counter condition shown in Figure 4, the apparent superdiffusive behavior of the particle appears as Brownian motion for most of the time, but is interrupted by short intervals when large steps are made to displace the particle to a new non-local position (i.e., pseudo-transport) as a consequence of the heavy-tailed likelihood to jump further than the Gaussian case. By combining these iid power-law conditions in both distance and time as shown in Figure 5, it can be observed that the particle is experiencing: moments of apparent Brownian motion, periods of extended rest, and apparent non-local transport within short periods of time.

**FIGURE 7:**
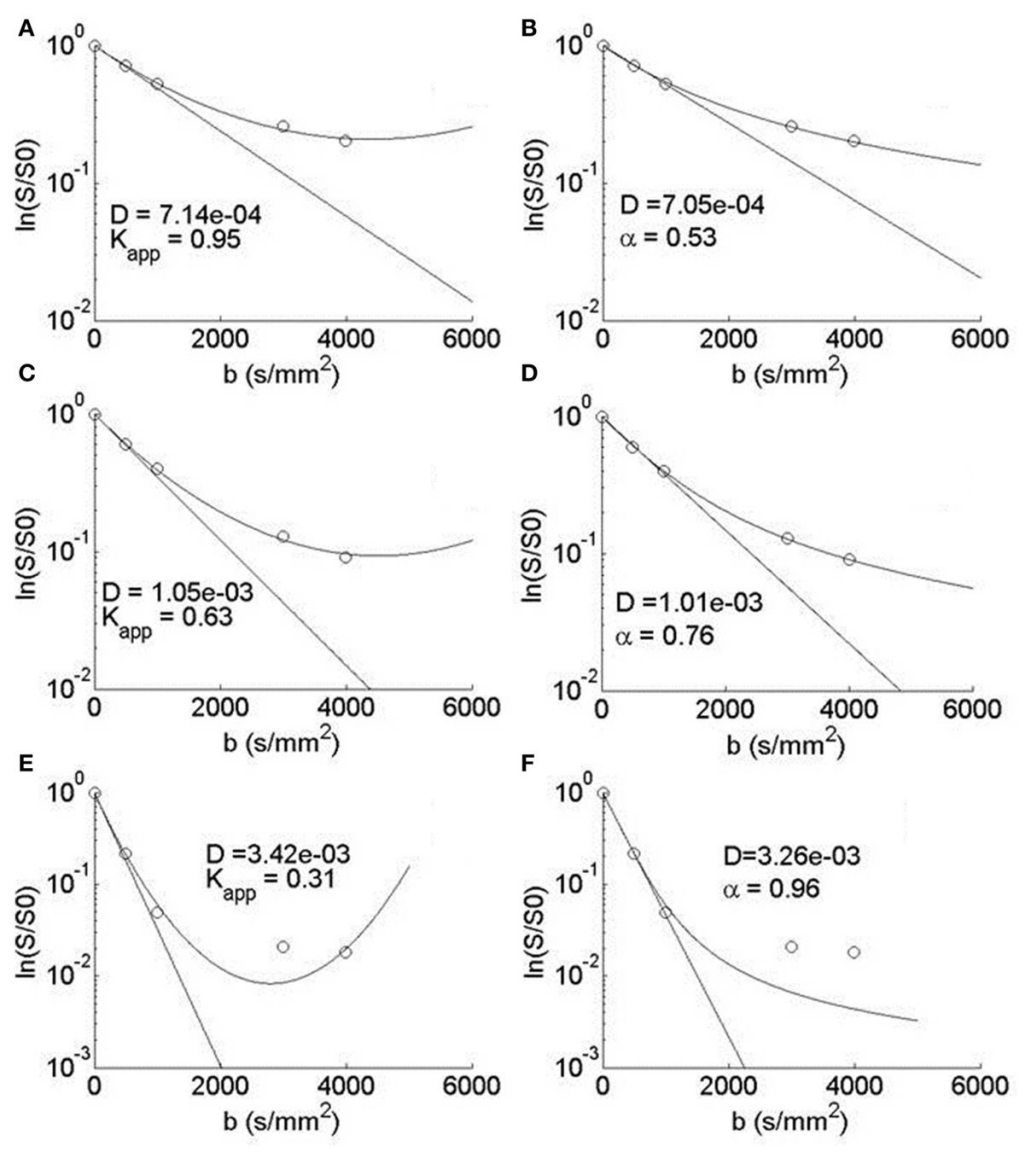
Semilog plots of typical voxel fits to Equation (24) in the (A) WM, (C) GM, and (E) CSF ROIs and Equation (22) in the (B) WM, (D) GM, and (F) CSF ROIs Each equation’s estimation of *D* (mm^2^/s) is shown to visualize a monoexponential fit and therefore the deviation from Gaussian diffusion.

In the diffusion MRI experiments, Table 1 shows the estimations of *α*, *K_MLF_*, *K_app_*, *D_MLF_* (estimated by Equation 24), *D_K_* (estimated by Equation 24), and *ADC* (estimated by Equation 4), for the ROIs circled in the parameter maps of Figure 6. The ischemic tissue in the right hemisphere of the patient’s brain (left side of the image) has an ADC value which is similar to the typical ADC found in the CSF. As these data were acquired ~ 2 years following onset, the IT has necrosed such that bulk ADC has increased to an unhindered value. Furthermore, the diffusion in the IT is close to Gaussian at all sampled length scales (*b*-values) as *α ~* 1, indicating a monoexponential behavior, which is also the case for the CSF. The trace values for ADC in the healthy WM and GM are ~ 1/3 of the values in the IT and CSF, with the WM possessing an overall slower diffusion than measured in the GM. As the scale in the *D* map in Figure 6 spans the range up to 3 × 10 ^−3^ mm^2^/s, the contrast between WM and GM is difficult to discern, however, in the *α* map the WM/GM contrast is clearly visible with the WM demonstrating more subdiffusive behavior compared to the GM. The *K_MLF_* map also has clearly visible GM/WM contrast and appears as a negative image with respect to the *α* map due to the nearly inverse relationship between *K_MLF_* and *α* in Equation (32). The *K_app_* map resembles similar GM/WM contrast in comparison to *K_MLF_*, albeit with a smaller dynamic range of values between the tissue types as summarized in Table 1, in which the average values in the WMROI are *K_MLF_* ~ 1.75 and *K_app_* ~ 0.99, whereas the average values in the GM ROI are *K_MLF_* ~ 0.75 and *K_app_* ~ 0.58. Figure 7 shows example fits of Equations (22) and (24) to the diffusion data in voxels of WM, GM, and CSF along with the respective estimations for *D* determined from each equation in order to visualize the deviations from monoexponential decay on a semilog scale.

## 4. Discussion

The one dimensional, single particle random walk simulations with varying statistical conditions provide an illustrative means to conceptualize the differences between Gaussian and non-Gaussian diffusion. Of course, in MRI measurements of diffusion in biological tissue, the spatial resolution of a voxel is mesoscopic and therefore contains populations of proton spins diffusing about a biological microstructure medium that has varying properties of heterogeneity and anisotropy. When the diffusion MRI experiment is designed to sample multiple length and/or time scales (i.e., *b*-values) in order to probe the tissue microstructure, it is reasonable to consider that the bulk diffusion properties are non-Gaussian due to the heterogeneity of the tissue, reflected by the presence of neurons, glial cells, and microvessels. The CTRW framework is rooted in the statistical properties of the diffusion process in order to offer a physical description of the random motion, and therefore, in the biological context, subdiffusion, for example, can be envisaged as proton spin populations trapped by the restrictions of neurons and glia (Figure 3) whereas superdiffusion, can be envisaged as proton spin populations carried by microvesicular transport (Figure 4). Typically, in MRI experiments, the diffusion weightings are selected such that *b* > 500 s/mm^2^ in order to minimize the influence of bulk flow in the diffusion measurement [42]. Therefore, in such experiments, it is appropriate to consider a model which allows for the sensitization of sub- and Gaussian diffusion behaviors, as proposed in Equation (22).

For diffusion MRI, it should be highlighted here that the direct relationship between the Fourier transform of the diffusion propagator and the diffusion weighted signal holds in the limit of the short pulse approximation (i.e., *δ* ≪ Δ). Preclinical imaging spectrometers are able to produce very small *δ*, however most clinical MRI systems are not equipped to meet this relationship, though new advances in gradient hardware have become available in the most advanced systems to approach *δ* ≪ Δ [23]. Therefore, the estimates of kurtosis in this study, using either Equations (24) and (32), approach the true kurtosis of the pdf for the diffusion propagator in the limit that *δ* ≪ Δ. In current standard clinical implementation of the diffusion-weighted SE-EPI experiment, MRI systems are programmable to select a particular *b*-value, however the specific values of *δ* and Δ are dependent on the magnitude of *b* as well as the available maximum gradient amplitude, *G*. As a best practice, one would want to tune *δ* and Δ, such that different time and length scales are probed in order to characterize the properties of the tissue microstructure. However, in this study, to achieve a maximum *b*-value of 4000 s/mm^2^, the scanner software set Δ = 41.2ms, *δ* = 30.6ms, which gives an effective diffusion time of 31ms. At this effective diffusion time, the sampled *q*-values are sensitive to length scales from ~ 2.8 – 7.9μm. Considering water is diffusing at a rate of ~ 0.6 – 1.0 s/mm^2^ in the WM and GM, the effective net water displacement ( $\text{using}\sim \sqrt{2Dt}$) is ~ 6 – 8μm. Therefore, depending on the microstructural heterogeneity and tortuosity in the biological tissue, the choices made to sample *q–*space can result in Gaussian diffusion (due to spatial averaging) for small values of *q* or exploit the non-Gaussian dynamics if *q* is large enough. As a result, higher order modeling of the diffusion attenuation signal (i.e., *α*, *K_MLF_*, and *K_app_*) are only “effective” values and are dependent on the measurement scheme of *q*, Δ, and *δ* [22, 24]. Ideally, one would tailor the specifics of the diffusion experiment to exploit the typical length scales of the particular biological structure of interest (e.g., axon, astrocyte, soma, etc.).

Utilizing the moment expansion of the time-fractional form of the MLF in Equations (29) and (31) provides an intimate link between kurtosis and subdiffusion through the Γ function and *α*. However, this link does not mean that Equations (22) and (24) are interchangeable, as each mathematical approach is a means to estimate the true kurtosis of the pdf for the diffusion propagator. In Table 1, if we compare the estimated values for *K_MLF_* and *K_app_* in the ROIs, there is a consistent trend in which the WM exhibits higher kurtosis than the GM, and the IT and CSF have the lowest values. However, *K_MLF_* estimates a wider dynamic range of kurtosis values for the ROIs compared to *K_app_* such that the ratio for the mean values of WM to GM is ~ 2.25 for *K_MLF_* and ~ 1.71 for *K_app_*. For the IT and CSF ROIs, the kurtosis estimated by *K_MLF_* is approximately half of the values as determined by *K_app_*. In consideration of the estimated values for the diffusion coefficient, both *D_MLF_* and *D_k_* are consistently greater than the *ADC* values (determined from the *b* = 1000 s/mm^2^ data) for all ROIs, however it appears that *D_K_* is more sensitive to overestimating the *ADC*.

The typical signal fits for WM, GM, and CSF voxels can be visualized in Figure 7. The parabolic form of Equation (24) is apparent in A, C, and E with the estimations in the WM and GM data fitted to the decreasing side of the parabola and the CSF data fitted to both the decreasing and increasing sides of the parabola. Clearly, for the *b* = 3000 and 4000 s/mm^2^ CSF data, the noise floor has been reached and both Equations (24) and (22) fitted the higher *b*-value data points poorly, however it appears that Equation (24) is more sensitive to converging to spuriously high kurtosis estimates in the ventricles and necrosed tissue due residual signal noise. Nevertheless, over a limited range of *b*-values, Equations (22) and (24) can produce similar, but not identical, estimations of kurtosis. As Equation (22) is not bounded by a maximum *b*-value, the MLF provides the opportunity to more completely sample *q*-space in order to more accurately estimate the true kurtosis of the diffusion propagator, which is an advantage as very high strength (300 mT/m) gradients have recently become available for diffusion imaging [23]. For example, by removing the *b* = 4000 s/mm^2^ data and fitting to Equation (24), estimates for *K_app_* in the WMROI increase by 11.8±4.9% and, in the GM ROI, *K_app_* increases by 5.8 ± 6.0%, in comparison to the estimates based on the fits including the *b* = 4000 s/mm^2^ data. In contrast, by removing the *b* = 4000 s/mm^2^ data and fitting to Equation (22), estimates of *K_MLF_* in the WM ROI only decrease by 4.2 ± 6.4% and, in the GM ROI, *K_MLF_* only slightly decreases by 1.2 ± 4.9%. Therefore, with respect to *K_app_* estimates using Equation (24), this suggests that not only is Equation (22) able to fit a wider range of *b*-values due to its complete monotonicity, but also that estimations (from Equation 32) of the true kurtosis are more stable and less susceptible to change due to one’s choice of the *b*-value sampling scheme in the diffusion experiment.

## 5. Conclusions

We have presented new, simplified fitting forms for the MLF as a three-parameter model in Equation (21) (for potential subdiffusion and superdiffusion) and a two-parameter model in Equation (22) (for potential subdiffusion only). The concepts of subdiffusion, superdiffusion, and Brownian motion have been simulated to illustrate the physical consequences of the movement of a particle in the statistical context of the CTRW theory, which potentially can have biological correlates in diffusion MRI. We have computed the kurtosis (*K_MLF_*) for time-fractional form of the MLF, which provides a context to relate subdiffusion (*α*) to diffusion kurtosis imaging (*K_app_*). Finally, this approach has been demonstrated on diffusion MRI measurements in the brain of a chronic ischemic stroke patient in which the true kurtosis of the diffusion propagator was estimated by fitting the data both to the MLF and the Taylor-series expansion about the argument for the logarithm of the exponential function. Current work is underway to apply Equation (32) to extract physical properties of tissue microstructure (e.g., surface-to-volume ratio). Due to limitations on scan time for the stroke patient protocol, this study only considered an isotropic analysis to compare *K_MLF_* with respect to *K_app_*. Future work will expand the scan protocol on healthy subjects to characterize the directional dependence of estimations for *α* and *K_MLF_* with respect to *K_app_* in tensor representations, which require additional diffusion weighting directions.

## Figures and Tables

**TABLE 1:** Mean and standard deviation of *α*, *K_MLF_*, *K_app_*, *D_MLF_*, *D_K_*, and *ADC* values for selected regions of interest (ROI) in the white matter (WM), gray matter (GM), ischemic tissue (IT), and cerebrospinal fluid (CSF) of a chronic ischemic stroke patient’s brain.

ROI	*α*	*K_MLF_*	*K_app_*	*D_MLF_*	*D_K_*	*ADC*
(A) WM	0.49 ± 0.04	1.75 ± 0.12	0.99 ± 0.07	0.72 ± 0.03	0.75 ± 0.03	0.68 ± 0.02
(B) GM	0.77 ± 0.03	0.75 ± 0.09	0.58 ± 0.05	0.97 ± 0.02	1.02 ± 0.02	0.93 ± 0.01
(C) IT	0.94 ± 0.03	0.18 ± 0.09	0.35 ± 0.03	3.12 ± 0.12	3.26 ± 0.11	2.97 ± 0.10
(D) CSF	0.97 ± 0.01	0.12 ± 0.03	0.29 ± 0.05	3.27 ± 0.11	3.46 ± 0.12	3.12 ± 0.08

The estimated diffusion coefficient values are reported with units × 10^−3^ mm^2^/s.
